# Processing of Feature Selectivity in Cortical Networks with Specific Connectivity

**DOI:** 10.1371/journal.pone.0127547

**Published:** 2015-06-17

**Authors:** Sadra Sadeh, Claudia Clopath, Stefan Rotter

**Affiliations:** 1 Bernstein Center Freiburg & Faculty of Biology, University of Freiburg, Feiburg, Germany; 2 Bioengineering Department, Imperial College London, London, UK; Neuroscience Campus Amsterdam, VU University, NETHERLANDS

## Abstract

Although non-specific at the onset of eye opening, networks in rodent visual cortex attain a non-random structure after eye opening, with a specific bias for connections between neurons of similar preferred orientations. As orientation selectivity is already present at eye opening, it remains unclear how this specificity in network wiring contributes to feature selectivity. Using large-scale inhibition-dominated spiking networks as a model, we show that feature-specific connectivity leads to a linear amplification of feedforward tuning, consistent with recent electrophysiological single-neuron recordings in rodent neocortex. Our results show that optimal amplification is achieved at an intermediate regime of specific connectivity. In this configuration a moderate increase of pairwise correlations is observed, consistent with recent experimental findings. Furthermore, we observed that feature-specific connectivity leads to the emergence of orientation-selective reverberating activity, and entails pattern completion in network responses. Our theoretical analysis provides a mechanistic understanding of subnetworks’ responses to visual stimuli, and casts light on the regime of operation of sensory cortices in the presence of specific connectivity.

## Introduction

Cortical neurons in early sensory areas are driven by inputs from a combination of feedforward and recurrent sources that eventually determine the selectivity of neuronal responses. In primary visual cortex (V1) of mammals, for instance, initiated by the seminal observations of orientation selective (OS) neurons by Hubel and Wiesel [1, 2], the contribution of feedforward and recurrent mechanisms has been thoroughly investigated [3–5]. Theoretical arguments were developed that the cortical recurrent network could be responsible for the emergence of orientation selectivity [6–8]. The results of inactivation experiments, on the other hand, have demonstrated that the thalamic input to cortical neurons are selective for orientation, even in absence of cortical interactions [9, 10].

The contribution of cortical recurrent networks to orientation selectivity has recently been reconsidered, in light of new experimental findings in rodents [11–13]. Given the salt-and-pepper arrangement of neuronal selectivities and absence of feature maps in rodent visual cortex [12], new questions have been raised with regard to specificity of recurrent connections [14]. Can orientation selectivity emerge in networks with random connectivity? Is feature-specific (FS) connectivity of the recurrent connections needed at all to obtain orientation selectivity in neuronal responses? It has in fact been reported that local cortical networks in rodent visual cortex of animals with visual experience have a non-random structure, with a higher probability of a synaptic connection between neurons of similar as compared to dissimilar preferred orientations [15–17]. Although orientation selective responses do exist, connectivity is random at eye opening, and FS connectivity only develops later [18, 19]. We hypothesized that the appearance of FS connectivity, for example induced by a Hebbian learning rule [18, 20], may therefore be responsible for the further enhancement and amplification of OS.

Consistent with this idea, it has recently been suggested by a number of studies that intracortical excitation can induce a linear amplification of the thalamic input [21–23]. The recurrent input has been reported to have the same feature specificity as the feedforward thalamic input, but with a larger (almost two-fold) amplitude. In addition, a moderate degree of pairwise correlations has also been demonstrated in mouse primary visual cortex [24]. It has been argued that this is a signature of functional organization of the circuitry, in absence of a smooth orientation map. Furthermore, a link between spontaneous and evoked activity has recently been documented in rodent visual cortex [25]. The spatial organization of stimulus-driven and spontaneous ensembles are very similar in mouse, reminiscent of previous reports in cat visual cortex [26, 27].

In order to gain some mechanistic understanding of these different, maybe separate effects, we used a large-scale modelling approach. We studied to which degree adding FS connections to an otherwise randomly wired recurrent network can account for these observations (namely, linear amplification of the thalamic input, emergence of pairwise correlations, and link between spontaneous and evoked activity). We found that FS connectivity between excitatory neurons does indeed lead to an amplification of the specific stimulus component, as a result of identically tuned input from within the network, and fully consistent with recent experiments [21–23]. This amplification is further accompanied by moderate to large pairwise correlations in the network [24, 28, 29], depending on the operating regime determined by the strength of FS connectivity. The operating regime also affects the link between spontaneous and evoked activity in the network.

Our large-scale network model elucidates the mechanisms underlying the phenomena emerging as a result of FS connectivity, and casts lights on the operating regime of cortical networks with specific connectivity. Moreover, it predicts rather unexpected functional properties of visual cortex networks as a result of FS connectivity. First, we observed that reverberating activity emerged within the subnetwork of active neurons preferring a similar orientation. Second, the network with FS connectivity was capable of pattern completion upon partial activation of feedforward input fibers. Our computational study of these emergent properties sheds light on their underlying network mechanisms.

## Materials and Methods

**Neuronal network**. Our model consists of a recurrent network of *N* = 5 000 leaky integrate-and-fire (LIF) neurons, of which *f* = 80% are excitatory and 20% are inhibitory [30]. The sub-threshold dynamics of the membrane potential, *V*_*i*_(*t*), of neuron *i* is described by the leaky-integrator equation

$$\begin{array}{c} \tau {\dot{V}}_{i}\left(t\right)+V_{i}\left(t\right)=RI_{i}\left(t\right). \end{array}$$

The current, *I*_*i*_(*t*), represents the total input to the neuron, the integration of which is governed by the leak resistance, *R*, and the membrane time constant, *τ* = 20 ms. When the voltage reaches the threshold at *V*_th_ = 20 mV, a spike is generated and transmitted to all postsynaptic neurons, and the membrane potential is reset to the resting potential at *V*_0_ = 0 mV. It remains at this level for short absolute refractory period, *t*_ref_ = 2 ms, during which all synaptic currents are shunted.

**Network connectivity**. Each neuron receives input from *ϵ*_exc_ = 20% of the excitatory population and *ϵ*_inh_ = 50% of the inhibitory population, sampled randomly. Inhibitory synapses are arranged to be *g* = 8 times more effective than the excitatory ones [31, 32], thus our networks are highly inhibition-dominated. This feature is motivated by dense connectivity of inhibitory neurons observed in different cortices [16, 33, 34], and functional reports for dominance of inhibition in the cortex [35, 36]. Postsynaptic currents are modeled as *δ*-functions, where the total current is delivered instantaneously to the postsynaptic neuron after each spike. Synaptic coupling is measured by the amplitude of the resulting postsynaptic potential (PSP), *J* = *RI*. The strength of connections made by excitatory neurons in the local network is *J*_exc_ = 0.2 mV; as a result, *J*_inh_ = −*gJ*_exc_ = −1.6 mV. Both excitatory and inhibitory PSPs decay exponentially with the membrane time constant *τ*, once activated.

In addition to input from local networks, neurons receive a background input from non-local recurrent sources. Each neuron receives an input from 5 000 non-local excitatory neurons with spontaneous firing rates of 1 spike/s, modeled as a stationary Poisson process. Synapses from non-local inputs have the same strength as local excitatory synapses, i.e. *J*_exc_ = 0.2 mV. Synaptic transmission delays are fixed at 1.5 ms throughout.

We implement feature specific (FS) connectivity in the network by changing the weights of already existing connections between neurons accordingly. The weight of a connection between the *j*th presynaptic neuron and the *i*th postsynaptic neuron, *w*_*ij*_, is modulated by a factor that depends on the cosine of the angular difference between the respective input preferred orientations of the two neurons

$$\begin{array}{c} \Delta w_{ij}=w_{ij}\;[1+\mu_{FS}\cos\left(2\left(\theta_{i}^{*}-\theta_{j}^{*}\right)\right)]. \end{array}$$

The parameter *μ*_*FS*_ describes the degree of FS modulation of the connectivity (FS Mod), with *μ*_*FS*_ = 0 corresponding to no modulation (no FS connectivity) and *μ*_*FS*_ = 1 corresponding to the case with the strongest possible modulation (zero weight for orthogonal POs).

**Network stimulation**. When the visual stimulus, i.e. an oriented elongated bar, is presented, feedforward input from the lateral geniculate nucleus (LGN) drives cortical neurons. The feedforward (thalamic) input to each neuron comprises an input from *N*_lgn_ = 50 LGN cells, each with a baseline firing rate of *r*_lgn_ = 20 spikes/s, using a synapse of strength *J*_lgn_ = 1 mV. This amounts to a baseline firing rate *s*_*b*_ = *N*_lgn_ × *r*_lgn_ = 1 kHz of the input to each neuron. The total input depends on the orientation of the stimulus, *θ*, and the preferred orientation (PO) of the neuron, θ*, according to a cosine function

$$\begin{array}{c} s\left(\theta ,\theta^{*}\right)=s_{b}\;[1+\mu \cos(2\left(\theta -\theta^{*}\right))]. \end{array}$$

The parameter *μ* is the modulation of the input tuning, which is set to *μ*_exc_ = 20% for excitatory neurons and *μ*_exc_ = 0% for inhibitory neurons in our simulations. Similar to the non-local recurrent input, the feedforward input is represented by a stationary Poisson process with rate *s*.

To measure the output tuning curves in numerical simulations, we stimulate the networks for 12 different stimulus orientations between 0° and 180°. For each stimulus orientation, the activity of the network is simulated and recorded for 5 trials, each lasting 1.5 s. The onset transient (150 ms) is removed from the analysis.

**Fig 1:**
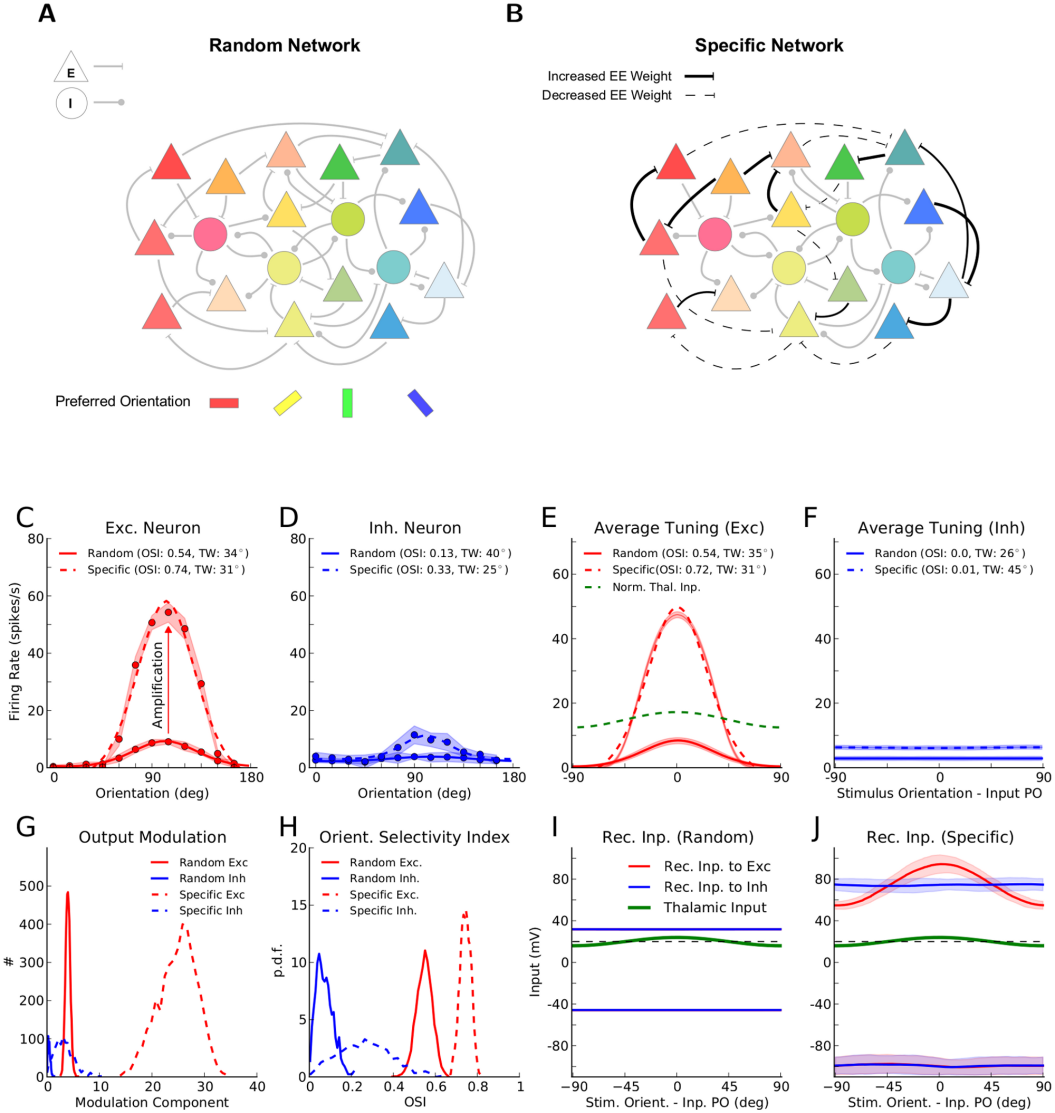
Orientation selectivity in networks with random or specific patterns of connectivity. (**A**) Random networks: connectivity between neurons is independent of the preferred orientation (PO; color coded) of the lumped feedforward input to each neuron. Triangles: excitatory neurons, circles: inhibitory neurons. (**B**) Networks with specific excitatory to excitatory (EE) connections: the weight of synapses between excitatory neurons is increased or decreased, if the two neurons have similar or dissimilar (close to orthogonal) input POs, respectively. (**C**) Tuning curve of a sample excitatory neuron with and without FS connectivity. For each orientation, the mean (circles) ± one standard deviation (shading) of the firing rate over 5 trials (1.5 s each) are plotted. The tuning width (TW) is extracted from a fit (see Materials and Methods) to the tuning curve (solid: random network, dashed: network with specific connectivity). The orientation selectivity index [38], OSI = 1−Circular Variance, is computed from the actual data points. (**D**) Same as (C) for a sample inhibitory neuron. (**E**) Mean (solid line) ± one standard deviation (shading) of excitatory output tuning curves, computed from the entire population, both for random and for specific networks. The green curve is the input tuning curve, normalized in amplitude to have the same mean as the average output tuning curve in the specific network. The OSI and TW are computed from the fit (solid: random network, dashed: specific network). (**F**) Same as (E) for the inhibitory population. (**G**) Distribution of the modulation component (second Fourier component, F2) of output tuning curves, for random (solid line) and for specific (dashed line) networks, respectively. (**H**) Distribution of the OSI for the population of excitatory (red) and inhibitory (blue) neurons, in random (solid line) and in specific (dashed line) networks, respectively. (**I**) Mean (solid line) ± standard deviation (shading) of recurrent inputs in the network with random connectivity, obtained from sample excitatory (red) and inhibitory (blue) neurons (50 each). (**J**) Same as (I) for a network with FS connectivity.

**Orientation selectivity**. To quantify orientation selectivity (as in e.g. Fig 1), we compute the Preferred Orientation and the Orientation Selectivity Index, OSI, of each neuron from its output tuning curve, *r*(*θ*), obtained in numerical simulations. We first compute the circular mean [37] of the firing rate measured at each orientation,

$$\begin{array}{c} R=\frac{\sum_{\theta }\;r\left(\theta \right)\exp\left(2i\theta \right)}{\sum_{\theta }\;r\left(\theta \right)}. \end{array}$$

The output preferred orientation is then extracted as the angle of the resultant, arg(*R*), and the length of it, ∣*R*∣, yields a global measure of orientation selectivity, OSI [38].

To each output tuning curve, *r*(*θ*), we fit a von Mises function:

$$\begin{array}{c} r^{\text{vM}}\left(\theta \right)=a+b\exp[k\cos(2(\theta -\phi ))-1], \end{array}$$

using a nonlinear least squares method.

From the best fitting function, the tuning width is extracted as

$$\begin{array}{c} \text{TW}=\frac{1}{2}\arccos[1+\frac{1}{k}\log(\frac{1+\exp(-2k)}{2})]. \end{array}$$

For each output tuning curve, *r*(*θ*), we also compute the baseline (zeroth Fourier, F0) and the modulation (second Fourier, F2) component. The baseline is obtained from the mean of the tuning curve over all orientations, and the modulation is obtained from the second Fourier component of the tuning curve.

**Fig 2:**
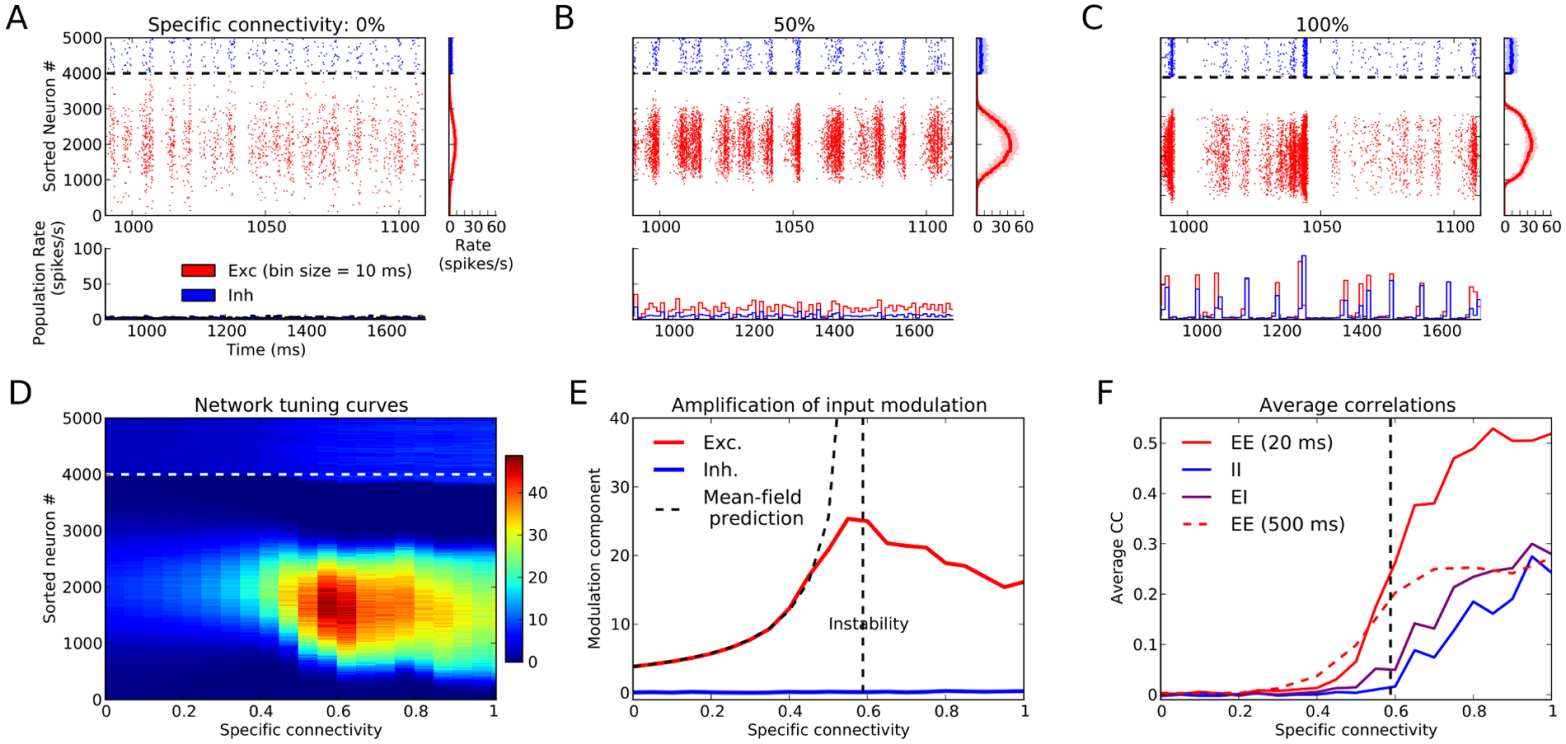
Tuning amplification in networks with different degrees of specific connectivity. (**A–C**) Raster plot of activity for networks with different specific connectivity in response to a stimulus with *θ* = 90°. Excitatory (red dots) and inhibitory (blue dots) neurons are sorted according to their input preferred orientation, respectively. The panels below each raster plot show the population firing rate of the corresponding networks, computed from the total spike counts in bins of size 10 ms. The panels on the right indicate the time-averaged firing rate of each neuron, extracted from 15 s of activity (“network tuning curve”). Shading: firing rate of all neurons, solid line: moving average using a window of width of 20 neurons. (**D**) Network tuning curves (color coded) for 20 different networks with specific connectivity (*μ*_FS_) between 0% and 100%. (**E**) Modulation (second Fourier, F2) components of average tuning curves in (D) for excitatory (red) and inhibitory (blue) neurons. The vertical dashed line indicates the critical value for the specific connectivity *μ*_FS_ at which the network becomes dynamically unstable. The dashed curve is a prediction for the amplification of output modulation derived from mean-field theory (see Materials and Methods). (**F**) Average pairwise correlations for networks with different degrees of specific connectivity. Pearson correlation coefficients (CC) of spike counts for bins of size 20 ms are computed for all pairs of randomly sampled neurons (100 excitatory and 100 inhibitory neurons). The average CC is plotted separately for excitatory-excitatory (EE, red), inhibitory-inhibitory (II, blue), and excitatory-inhibitory (EI, purple) correlations. Correlation coefficients for larger or smaller bin sizes show the same overall trend (S4 Fig). Average EE noise correlations for a very large bin size (500 s) are shown by the dashed line here (see Materials and Methods, for details).

**Pairwise correlations**. Pairwise correlations (in Fig 2F and S4 Fig) are computed from single-neuron spike counts in small bins. For a pair of neurons with spike count vectors *n*_*i*_ and *n*_*j*_ (computed from 15 s total simulation time), the correlation is obtained as the correlation coefficient of the respective spike counts

$$\begin{array}{c} {\text{CC}}_{ij}=\rho_{n_{i},n_{j}}=\frac{\text{cov}(n_{i},n_{j})}{\sigma_{n_{i}}\sigma_{n_{j}}}=\frac{E[\left(n_{i}-\mu_{n_{i}}\right)\left(n_{j}-\mu_{n_{j}}\right)]}{\sigma_{n_{i}}\sigma_{n_{j}}} \end{array}$$

where *μ* and *σ* denote vector of the means and standard deviations of the spike counts.

Pairwise correlations are also computed, in the same fashion, for longer bins (the dashed line in Fig 2F and panels D–F in S4 Fig). This is computed from spike counts of neurons for 200 trials of 500 ms duration each. All neurons with firing rates higher than 1 spikes/s are considered in this analysis. The correlation coefficient computed here is similar to computing noise correlations as the product of z-scores between a pair summed across all trials (see e.g. [24]).

**Linear gains**. The spectrum of eigenvalues of the weight matrix shown in S3 Fig is normalized according to the linearized input-output gain, *ζ*, for individual neurons: Each synaptic weight is replaced by an effective weight *J*_eff_ = *ζJ*. This gain is obtained by linearizing the mean response rate of a LIF neuron [39]

$$\begin{array}{c} r=f\left(\mu ,\sigma \right)={\left[t_{\text{ref}}+\tau \sqrt{\pi }\int_{{\tilde{V}}_{0}}^{{\tilde{V}}_{\text{th}}}e^{u^{2}}\left(1+\mathrm{erf}\left(u\right)\right)\;du\right]}^{-1}, \end{array}$$

where *μ* and *σ* are mean and standard deviation of the current fluctuation input to the neuron, respectively, ${\tilde{V}}_{\text{th}}=(V_{\text{th}}-\mu )/\sigma$ and ${\tilde{V}}_{0}=(V_{0}-\mu )/\sigma$, and erf is the error function.

The linearization is performed about the baseline state, *s* = *s*_*b*_. Under this condition, and assuming homogeneous firing rates in the network (mean-field ansatz) and neglecting correlations, mean and variance of the input can be written as [40]

$$\begin{array}{ccc} \mu_{b} & = & \tau \;[J_{\text{lgn}}s_{b}+J_{\text{exc}}r_{b}N\left(\epsilon_{\text{exc}}f-\epsilon_{\text{inh}}g\left(1-f\right)\right)],\\ \sigma_{b}^{2} & = & \tau \;[J_{\text{lgn}}^{2}s_{b}+J_{\text{exc}}^{2}r_{b}N\left(\epsilon_{\text{exc}}f+\epsilon_{\text{inh}}g^{2}\left(1-f\right)\right)]. \end{array}$$

Here, *r*_*b*_ is the mean neuronal firing rate when all neurons are driven by the same baseline input *s*_*b*_ without any modulation *r*_*b*_ = *f*(*μ*_*b*_,*σ*_*b*_). We compute this rate numerically by solving the respective self-consistency equations [40].

To obtain the linear gain we consider a small perturbation the input, *δs*, and compute the resulting output perturbation, *δr*,

$$\begin{array}{c} \delta r=f\left(\mu \left(s_{b}+\delta s\right),\sigma \left(s_{b}+\delta s\right)\right)-f\left(\mu \left(s_{b}\right),\sigma \left(s_{b}\right)\right). \end{array}$$

The linear gain is then given by *J*_lgn_ *ζ* = *δr*/*δs*. In S3 Fig, *ζ*_*s*_ was obtained from choosing *δs* of the same size as the input modulation, *δ*_*s*_ = *s*_*m*_ = *μs*_*b*_.

**Stability criterion for networks with specific connectivity**. We aim to determine when the eigenvalues responsible for the modulation component in the input (here, the eigenvalues with the most positive real parts, see S3B Fig) become unstable. The modulation eigenvalue, *λ*_*m*_, is a result of feature specific recurrent synaptic connectivity in the network. If neurons are sorted according to their preferred orientations, the weight matrix, *W*, can be approximated as a circulant matrix (for the network with all-to-all connectivity, *W* is a circulant; here, for our random networks, we approximate the matrix as a circulant matrix corrupted with noise). In the mean-field approximation, therefore, the operation of *W* on an input vector, *s*

$$\begin{array}{c} r=Ws \end{array}$$

can be written in terms of a convolution

$$\begin{array}{c} r=w*s, \end{array}$$

where *w* is the first row of *W*. The convolution corresponds to a product in the Fourier domain

$$\begin{array}{c} \hat{r}=\hat{w}\;\hat{s}. \end{array}$$

We are interested here in the second Fourier component, which is an eigenmode of the network, and therefore write

$$\begin{array}{c} r_{m}=w_{m}s_{m}=\lambda_{m}s_{m}. \end{array}$$

The modulation of the output is now a product of two modulations: a modulation in the cosine input is multiplied by the modulation component of the cosine connectivity.

We can therefore compute *λ*_*m*_ as the modulation component of the cosine tuning of each row of the weight matrix. The parameter *μ*_FS_ determines the degree of modulation, and given the parameters of network, the resultant value can be computed as

$$\begin{array}{c} \lambda_{m}=\frac{1}{2}\mu_{\text{FS}}fN\epsilon_{\text{exc}}\zeta J_{\text{exc}}, \end{array}$$

if only the excitatory connections are specific (S3B Fig). A dynamic instability sets in for the critical $\mu_{\text{FS}}^{*}$ for which Re(*λ*_*m*_) = 1, and we obtain

$$\begin{array}{c} \mu_{\text{FS}}^{*}=\frac{2}{fN\epsilon_{\text{exc}}\zeta J_{\text{exc}}}. \end{array}$$

This is the vertical dashed line indicated in Fig 2E.

The output activity of the network in response to input stimuli is obtained by considering the full *N*-dimensional dynamics of neuronal firing rates. Assuming linear operation of the network, the linear rate equation in the stationary state leads to the following stimulus-response relation

$$\begin{array}{c} r={\left(𝟙-W\right)}^{-1}s. \end{array}$$

The network operator *A* = (𝟙 − *W*)^−1^ is obtained by applying an analytic function to the weight matrix *W*, and hence the corresponding modulation eigenvalue of *A*, $\lambda_{m}^{\prime }$, can be obtained by applying the same analytic function

$$\begin{array}{c} \lambda_{m}^{\prime }={\left(1-\lambda_{m}\right)}^{-1}. \end{array}$$

This is the factor with which the network amplifies any modulation in its input, as long as its operation is linear. Output modulation is therefore amplified by the network with FS connectivity, relative to its baseline value, $r_{m}^{0}$, in absence of FS connectivity, according to

$$\begin{array}{c} \frac{r_{m}}{r_{m}^{0}}=\frac{1}{1-\frac{1}{2}\mu_{\text{FS}}fN\epsilon_{\text{exc}}\zeta J_{\text{exc}}}. \end{array}$$

The dashed curve in Fig 2E is derived from this relation. To some extent, it explains the non-linear dependency of the amplification gain on the connectivity parameter.

## Results

### Intracortical amplification of feedforward tuning

Using large-scale simulations of inhibition-dominated cortical networks, we studied the dynamic consequences of adding specificity to recurrent connections. Concretely, we considered enhanced connectivity between neurons with similar preferred orientations (Fig 1A, 1B). We found that such specific recurrent synaptic interactions between excitatory neurons indeed generally amplify the tuned output responses (Fig 1C–1H). This is beyond the orientation selectivity that can already arise in random networks in absence of specific interactions [31, 41, 42] (S1 Fig). Such an enhancement of orientation selectivity has in fact been reported developmentally after eye opening in experimental studies [18, 43]

Consistent with recent experimental findings [21–23], the excitatory recurrent input to excitatory neurons from within the network has a similar tuning as the feedforward input, only with a larger amplitude (Fig 1J and S2 Fig). Such tuned recurrent inputs are conspicuously lacking in random networks (Fig 1I). Also, recurrent excitatory input to inhibitory neurons, and recurrent inhibitory input to both excitatory and inhibitory neurons are on average untuned (Fig 1J), as we have not implemented specific connectivity in those connections. In both random and specific cases (Fig 1I and 1J, respectively), however, the untuned components of feedforward and recurrent contributions cancel each other, and only the tuned component can trigger output spikes.

### Regimes of intracortical amplification

We next asked how the properties of orientation selectivity depend on FS connectivity. It might be expected that orientation selectivity is enhanced as FS connections are enhanced. We therefore systematically increased the strength of FS connectivity in our networks, while keeping all the other parameters including the structure of connectivity the same (Fig 2).

Sample spiking activity, population responses and network tuning curves for three networks (from random to highly specific) are shown in Fig 2A–2C, respectively. Network tuning curves extracted from all simulated networks covering a range of FS connectivity are shown in Fig 2D. The tuning of output tuning curves is quantified by their respective modulation (second Fourier, F2) components. This is shown in Fig 2E, separately for excitatory and inhibitory neurons, respectively.

Surprisingly, our results show a non-monotonic dependence of tuning amplification on FS connectivity, where intermediate levels of specificity lead to the strongest amplification (Fig 2D and 2E). Further mathematical analysis revealed that the reason for this non-monotonic dependence is the emerging instability of the eigenmode associated with modulation (see Materials and Methods and S3 Fig; hereafter, we refer to this kind of instability as “spectral instability”). Indeed, at the edge of spectral stability (Fig 2E) the network displays the highest amplification; beyond that point, increasing specificity cannot enhance the output responses anymore.

Note that neurons are never reaching their maximal firing rate, which is 500 spikes/s given the refractory period of 2 ms for the neuron model used here. In fact, we never observed runaway firing rates in our networks. Therefore, a saturation nonlinearity is not the reason for the apparent stability of the system. Instead, nonlinear recurrent interactions in the network are responsible for this stabilization.

An explanation of this network operation can be provided as follows. When the modulation components of output tuning curves are comparable to their baseline components on average, very limited rectification occurs in the network, and the input-output transformation of tuning curves is mainly linear (see [32] for details). When the modulation component becomes too large, a rectifying nonlinearity sets in for very low firing rates. As a result of this nonlinearity, our cosine input tuning curves are not transformed to cosine output tuning curves, because the linearity of network operation is violated. Now the orthogonality of baseline and modulation components, which holds in the linear regime of operation [31], is not effective any more. As a consequence, the modulation component cannot be amplified by the network beyond any bound: for very large output modulations, the negative part of the cosine tuning curve is rectified and hence it obtains a non-zero mean value. This now leads to a projection over the common-mode of the network, which is in turn suppressed in inhibition-dominated networks [31]; this means that the output rectified modulation becomes suppressed by the strong negative feedback which was only acting on the uniform mode (the baseline component of the input) before (for a similar observation, see [32]). This in effect yields a suppressive mechanism which controls the amplification of modulation for very large values and hence nonlinearly stabilizes the network. This is also the reason for the discrepancy of the linear prediction and the actual amplification close to the critical value of FS connectivity, already before entering the region with spectral instability.

In the regimes where the nonlinear mechanisms are not dominant, our theoretical analysis fully explains how feedforward and recurrent parameters interact in generating the output selectivity. According to this analysis, the output modulation in the absence of recurrent specificity ($r_{m}^{0}$) is amplified by feature-specific connections by a factor of $\frac{1}{1-\frac{1}{2}\mu_{\text{FS}}fN\epsilon_{\text{exc}}\zeta J_{\text{exc}}}$ (see Methods). Here *μ*_FS_ is the strength of feature-specific connectivity (ranging from 0 for random connectivity to 1 for the maximally specific network), *N* is the total number of neurons in the network and *f* is the fraction of excitatory neurons, *J*_exc_ is the absolute weight of excitatory connections and *ζ* is the linearized gain of leaky integrate-and-fire neurons about the baseline operating point of the network.

Already from here, several questions about the amplification of orientation selectivity in our networks can be answered: first, what is important for this amplification is the product of recurrent excitation (*fNϵ*_exc_) and its feature-specificity (*μ*_FS_). That is, the same degree of feature-specific connectivity in a more weakly (strongly) connected network leads to a smaller (larger) amplification. Furthermore, the total amplification obviously depends on how tuned the feedforward input is, as the recurrent specificity is only multiplying the output tuning existing in the non-specific case, which is in turn directly affected by the tuning of the feedforward input (see [31, 41, 42]).

It can also be asked how this amplification scales with network size, especially for infinitely large networks. To answer this, we can evaluate the critical value of feature-specificity at which the network becomes spectrally unstable: $\mu_{\text{FS}}^{*}=\frac{2}{fN\epsilon_{\text{exc}}\zeta J_{\text{exc}}}$. Let us first assume that the linear gain, *ζ*, is 1, which would be the case for perfect integrate-and-fire neurons [32]. For infinitely large networks, *N* → ∞, if the weights are not rescaled and the connection probability is the same, $\mu_{\text{FS}}^{*}$ would be zero, that is the network becomes unstable with very small feature-specificity.

To avoid that, rescaling the connection probability or synaptic weights becomes necessary. Another solution is to balance the specific excitatory connectivity with specific inhibitory connections. However, there is no biological evidence so far for orientation-specific connectivity of inhibitory neurons, as they are reported to densely connect to their local neighborhood [16], which, in rodents, comprises neurons with heterogeneous preferred orientations. Another solution, which is consistent with unselective inhibitory connections, is that inhibitory neurons are not balancing the specific input to the neurons, but affecting the linear gains, *ζ*. This way, it is possible that a stronger inhibitory feedback, presumably as a result of synaptic plasticity of inhibitory-excitatory connections, decreases *ζ*, which in turn increases the $\mu_{\text{FS}}^{*}$ and stabilizes the network in response to modulated inputs. For networks of leaky integrate-and-fire neurons, in particular, *ζ* can be computed [44]; it is therefore possible to compute the exact dependence of the $\mu_{\text{FS}}^{*}$ given any combination of parameters for the network.

### Pairwise correlations in the network

Amplification of orientation selectivity in FS networks is accompanied by an increase in pairwise correlations. Unlike the amplification itself, however, this increase has a monotonic dependence on FS connectivity, and correlations increase in the network as FS connectivity becomes stronger (Fig 2F and S4 Fig).

Notably, the largest correlations arise in the spectrally unstable regime (Fig 2F). For spectrally stable networks, average correlations are rather weak, consistent with an asynchronous-irregular (AI) state of network activity [40]. Population responses deviate more from AI-type network activity, however, in the spectrally unstable regime, and they approach a highly synchronous state, characterized by very large temporal fluctuations of the population activity (Fig 2C). Emergence of large correlations indeed starts when networks approach the edge of spectral stability, as has been demonstrated and analyzed before [45].

The regime of recurrent amplification can therefore be summarized in terms of three regimes of (a) low, (b) medium and (c) high FS connectivity: (a) shows very little tuning amplification and close-to-zero pairwise correlations; (b) exhibits maximum amplification and a mild degree of correlations (noise correlation close to 0.1); (c) entails a decreasing amplification, while at the same time pairwise correlations still increase.

### Spontaneous and evoked patterns of activity

**Fig 3:**
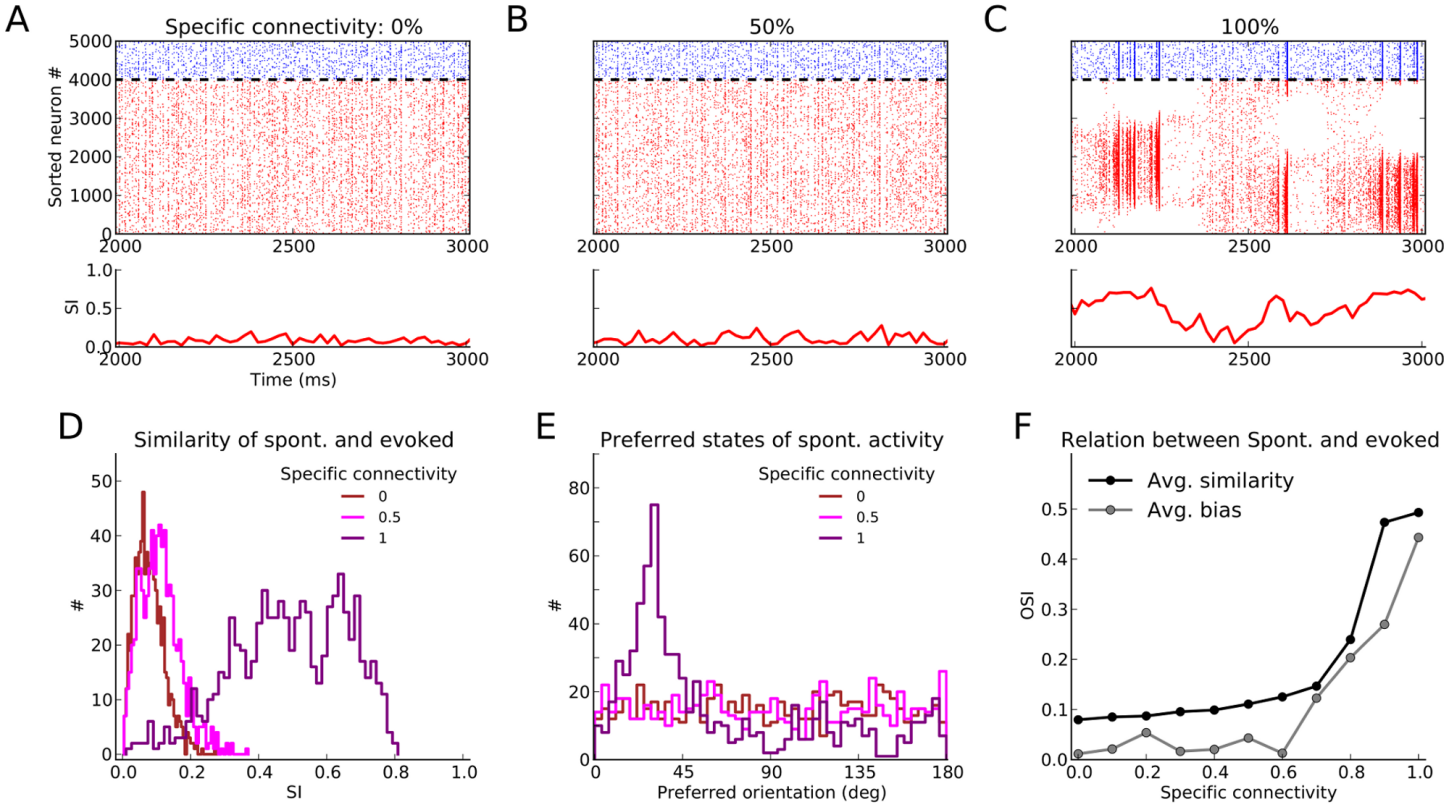
Spontaneous activity patterns. (**A–C**) Raster plot of spontaneous activity for networks with different degrees of specific connectivity. The similarity between spontaneous activity and patterns of activity evoked by oriented stimuli is quantified by a similarity index (SI). It is taken as the magnitude of the complex number $R^{\text{sp}}=\frac{\sum_{j}\;r_{j}^{\text{sp}}\text{exp}(2i\theta_{j}^{*})}{\sum_{j}\;r_{j}^{\text{sp}}}$, where $r_{j}^{\text{sp}}$ is the average firing rate of excitatory neuron *j* in bins of size 20 ms, and $\theta_{j}^{*}$ is the input preferred orientation of the neuron. Other conventions are as in Fig 2. (**D**) Distribution of SI of spontaneous activity extracted from a simulation of duration 30 s for three networks shown in (A–C). (**E**) The angle of *R* ^sp^ indicates the preferred orientation of the population activity in each time window. The distribution of this “spontaneous” tuning of population activity is plotted here for the three networks shown in (A–C). (**F**) The average SI of spontaneous activity, computed from distributions as in (D), is plotted for 11 networks ranging from completely random to highly feature-specific (black dots). To quantify the bias in the preferred orientation of the population activity in each network, the selectivity of the distributions of preferred orientations as in (E) are computed (Avg. bias, gray). It is computed as 1−Circular Variance of the distribution in (E).

We then asked the question of how spontaneous patterns of activity are influenced by FS connectivity (Fig 3). Inspecting the raster plots of activity in the three regimes of low, medium and high FS connectivity (Fig 3A–3C) reveals that the most structured pattern of spontaneous activity is observed in the regime of high FS connectivity. Temporal patterns of spontaneous population activity show structured activity, reminiscent of the patterns evoked by an oriented stimulus.

To quantify the similarity between spontaneous and evoked activity patterns, we computed a similarity index (SI) in bins of width 20 ms (Fig 3A–3C, lower panels). The most similar responses are again observed in the spectrally unstable regime (Fig 3C). This spontaneous activation of evoked responses in absence of a specific stimulus exhibits slow transitions between different preferred orientations (i.e. different points on the line attractor of the network; see [46] for similar observations).

To obtain further insights on the patterns of spontaneous activity in each regime, we plotted the distribution of similarity index and the preferred orientation of population activity for all three networks (Fig 3D, 3E). Although the most similar spontaneous activity is obtained for the spectrally unstable network, the network with medium FS also shows some enhancement of SI as compared to the random network (Fig 3D). However, in contrast to the spectrally stable networks with uniform distributions of preferred orientations, the spectrally unstable network does not represent all orientations uniformly in its spontaneous activity. Some orientation-selective responses are over-represented, which is a result of the specific realization of the random connectivity matrix, reflected by the eigenvalue spectrum of the weight matrix (see S3B Fig).

A bias for certain preferred orientations in spontaneous activity can be quantified by the selectivity index of the distribution of preferred orientations, shown in Fig 3E. A flat distribution with no bias whatsoever would return 0, whereas a distribution of spontaneous activity concentrated in a single preferred orientation would return 1. All networks in the spectrally stable regime have a uniform distribution of preferred orientations, while transition to instability renders the spontaneous activity of networks more biased towards specific evoked states as FS connectivity increases (Fig 3F, gray line). Thus, the increase in the SI of population activity for these networks (Fig 3F, black line) comes at the price of not visiting all states with the same frequency. In the intermediate regime, however, the SI of spontaneous activity is increasing, while the bias remains at low levels.

### Emergence of reverberating activity and pattern completion

**Fig 4:**
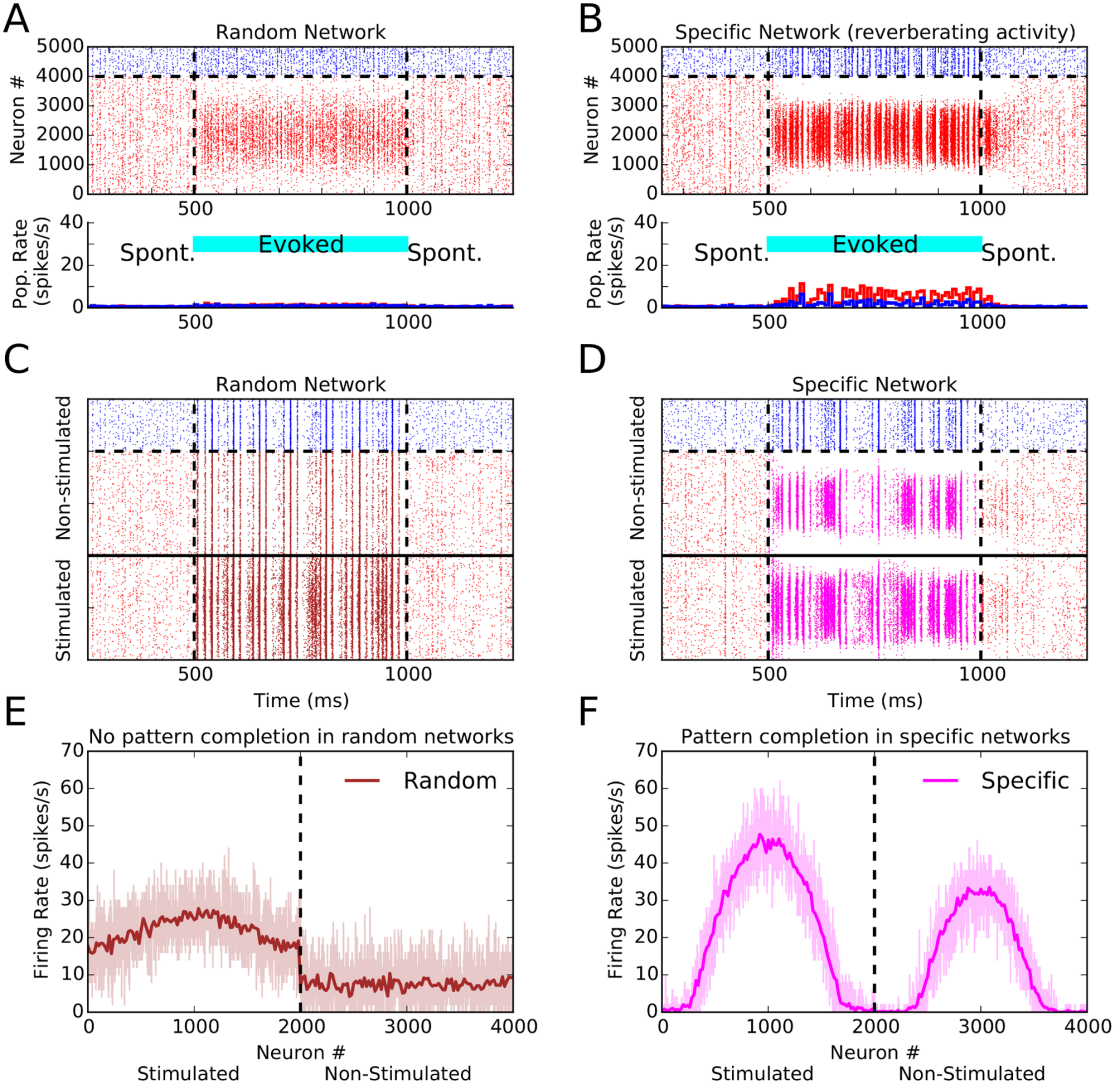
Reverberating activity and pattern completion in networks with specific connectivity. (**A**) Raster plot of a random network with non-specific stimulation (“spontaneous activity), and in response to a stimulus of orientation 90° (evoked activity) applied from 500 ms to 1000 ms (cyan bar). The bottom panel shows population firing rates computed in bins of size 25 ms. Excitatory neurons are red, inhibitory neurons are blue. (**B**) Same as (A), for a network with specific connectivity (specificity 50%). (**C, D**) Same as (A, B), but only a fraction of excitatory neurons is stimulated. The excitatory population is split into two halves, and only the first half (neurons below the solid line) sees the oriented stimulus; the remaining neurons receive only background input. (**E, F**) Network tuning curves (similar to Fig 2) extracted from the spiking activity of stimulated and non-stimulated excitatory neurons during the evoked state, for random and specific networks, respectively. In the random network, the non-stimulated fraction shows non-selective activity. In the network with FS connectivity, however, both stimulated and non-stimulated population show orientation selective responses.

We focused so far on how FS connectivity can lead to different regimes of tuning amplification, activity correlation, and spontaneous activation of specific activity patterns. However, additional functional properties can result from FS connectivity. In particular, we observed the emergence of reverberating activity, and pattern completion (Fig 4).

First, we observed that upon turning off an oriented stimulus networks with FS connectivity can show persistent activation for a brief period of time (50–100 ms; Fig 4B). As a result of feature-specific connectivity, some reverberation of activity emerges between neurons with preferred orientations close to the orientation of the stimulus. Such network-based short-term memory is lacking in the random network, and the evoked response quickly vanishes as soon as the oriented stimulus is turned off (Fig 4A).

Second, the network with FS connectivity was capable of pattern completion. Specifically, if only a fraction of excitatory neurons was stimulated with an oriented stimulus, the network with specific connectivity was capable of activating the non-stimulated neurons, with a similar pattern compared to that obtained by direct stimulation (Fig 4D, 4F). In a random network, there are no such spontaneous patterns to begin with (Fig 4C, 4E).

## Discussion

Using numerical simulation and computational analysis of large-scale spiking networks, our study demonstrated how the refinement of connectivity during development contributes to the processing of orientation selectivity in cortical networks. It provided a mechanistic link between FS connectivity and processing of orientation selectivity in a biologically realistic network model, fully consistent with recent experimental findings in the rodent visual cortex [21, 22]. Our mean field and spectral radius analysis could in fact provide a computational account of orientation selectivity in realistic model cortical networks operating in different regimes of FS connectivity.

An important feature of our networks was the dominance of inhibition in recurrent connectivity. This yields a selective suppression of the common-mode, which leads to an enhancement of output selectivity and contrast invariance of output tuning curves [31]. Inhibition also determines the effective linear neuronal gains in the networks, as the operating point about which the system is linearized is determined by the fixed point of the network (resulted from balance of excitation and inhibition) in response to the homogeneous input [44]. The inhibition-dominance of connectivity, in turn, leads to the balanced state in terms of network dynamics, where the net excitatory input to a single neuron is more than its threshold and only a comparable negative feedback from the network keeps the membrane potential below threshold and allows the neuron to operate in a fluctuation-driven regime of activity (as it is shown in Fig 1 and S1 and S2 Figs).

In contrast to previous modeling studies which suggested the marginal regime of recurrent connectivity is best for orientation selectivity [6], our modeling study suggested that there is an optimal value for feature-specific connectivity of the excitatory population. Beyond that, FS is detrimental to tuning amplification. Our model thus casts light on the operating regimes of intracortical amplification in the presence of specific connectivity. We found that there is a linear regime of amplification, where the results of a mean-field analysis of the network dynamics in the asynchronous-irregular, AI, state holds [40, 47], and where the network shows the highest amplification of the feedforward tuning. Beyond that optimal value of FS connectivity, the output selectivity decreases again, as a result of nonlinear interactions within the network. Notably, the same nonlinear mechanisms protect the network from global instability (involving runaway excitation). Thus, the optimal regime of tuning amplification is at the edge of instability of the AI state, just before the eigenmode corresponding to the modulation component would become linearly unstable (cf. [47]). In contrast to the conclusion of [47], therefore, our results suggest the stable AI state as a more suitable regime for sensory processing.

In this optimal regime, we observed a moderate increase of correlations (Fig 2F and S4 Fig), despite the network being spectrally stable. A comparable level of noise correlations has in fact been reported recently in mouse visual cortex [24]. This result is also consistent with larger pairwise correlations reported recently in supra- and infra-granular layers of monkey primary visual cortex [28, 29], assuming that FS connectivity is emerging in those layers as a result of Hebbian learning. We therefore hypothesize that the emergence of pairwise correlations might be considered as a signature of FS connectivity to the degree to which it implies reaching the edge of spectral stability [45]. In light of this hypothesis, it will be interesting to see whether the close-to-zero correlations in the input layers of monkey V1 [28, 29] reflect the lack of FS connectivity in those layers. Also, as FS connectivity is not present at eye opening in mice [18], we predict that only very low pairwise correlations should be observed at this stage, with moderate correlations appearing only later during development, coinciding with the emergence of FS connectivity [16–19].

Note that, although correlations are observed in the activity of our networks, they were not necessary, and not detrimental, for the emergence of tuning amplification in the linear regime. In fact, the mean-field theory employed here was developed under the assumption that neurons receive uncorrelated spike trains (see Methods and Materials). This theory was nevertheless generating an excellent approximation of the mean amplification of the network, and the transition to spectral instability (Fig 2E). The success of our model, therefore, shows that mechanisms underlying the amplification of feedforward tuning in the linear regime do not include correlations across neurons; tuning amplification and correlations both emerge as a result of network dynamics, but they do not seem to be linked together causally.

As opposed to the low or moderate degree of correlations in the regimes of stable dynamics, relatively large pairwise correlations were observed in the spectrally unstable regime (Fig 2F). That is, in presence of FS connectivity, the amplification gain of the network decreases, and pairwise correlations simultaneously increase. The former effect decreases the signal-to-noise ratio, and the latter effect compromises the decoding of sensory information [48]. In contrast, the regime with low FS connectivity shows only very small pairwise correlations. This enhances the stimulus encoding, but limits the network in amplifying the sensory signal. The intermediate regime appears to provide a useful trade-off between information content of the population code and signal-to-noise ratio.

A similar trade-off can be reported for the patterns of spontaneous activity (Fig 3F). Networks with very large FS showed the most selective spontaneous patterns, resembling the evoked responses of the network best. This was, however, compromised by a non-uniform representation of orientation patterns in the spontaneous activity (see [44] for a similar observation). The intermediate regime, in contrast, showed only a moderate enhancement of selectivity of spontaneous patterns, but without introducing a bias toward a specific preferred state (Fig 3F). Another advantage of the intermediate regime is the fast switching between different states, in contrast to the very slow time scale of transitions between network states in the spectrally unstable regime (see also [46]).

Note that it would be possible to further enhance the similarity of the spontaneous and evoked patterns in the intermediate regime by including NMDA synapses with slower kinetics into the model, similar to a previous modeling study [49]. Implementing this mechanism in a biologically more detailed model would ensure that the neuronal ensembles which activate together during spontaneous activity become even more pronounced and remain more active. Whereas such ensembles would be random groups of neurons which are more connected together by chance in a random network, the spontaneously active subnetworks would become feature-specific in networks with FS connectivity. Now, a pronounced functional map of orientation selectivity determines co-active ensembles in spontaneous activity and makes them similar to the evoked ones.

A biologically more realistic version of our model should also account for two further features. First, we neglected heterogeneity in synaptic connections, as the number and strength of synapses were the same for all networks, and only the strength of synapses was modified based on FS of pre- and post-synaptic neurons. A more biologically realistic network with heterogeneous connectivity (including heterogeneity in the number and strength of synapses, heterogeneity in FS connectivity, and heterogeneity in the balance of excitation and inhibition) leads to more realistic responses. However, our main findings here also hold under such conditions, and do not change qualitatively (results not shown). Second, we only studied networks in which only the excitatory population underwent a change in synaptic connectivity. It is possible, however, that during development, excitatory to inhibitory or inhibitory to excitatory connections also change. Potentiation of inhibition as a result of such a plasticity may well ensure the stability of the modulation eigenmode, preventing the network from entering the spectrally unstable regime. As a result of potentiation of inhibitory connections the firing rate of excitatory neurons can also decrease. This might lead to moderate firing rates which seem to be more compatible with experimental results in rodents. It remains, therefore, an interesting topic for further studies how concomitant changes in the weights of excitatory and inhibitory connections affect network dynamics and neuronal tuning curves.

In our networks here with FS excitatory connections, we also found two further emergent properties. First, we observed pattern completion in our networks: when only half of the excitatory population was stimulated, networks with FS connectivity responded as if the whole stimulus pattern was present. We also observed an emergence of reverberating activity as a result of specific connectivity, where orientation selective population activity persisted for a few tens of millisecond after removal of the visual stimulus. Such a persistent activity has indeed been reported in cats [50], with time constants very similar to those reported here. It is therefore interesting to see whether a similar effect exists in mouse visual cortex. As feature-specific connectivity is absent at eye opening, the prediction is again that reverberating activity only emerges later during development, as soon as a significant degree of FS connectivity is established [18]. It is also tempting to speculate about the biological function of this reverberating activity. It might be crucial for creating association in time, and instrumental to solve the problem of temporal feature-binding. Using analytical and computational tools, we therefore provide a very-much needed framework to understand the role of FS connectivity in visual processing in particular, and sensory processing in general.

## Supporting Information

S1 Fig Orientation selectivity in inhibition-dominated networks with random connectivity. (**A**) Spikes elicited by a sample excitatory neuron in response to stimuli of different orientations, offered to the network in 5 independent trials. (**B**) Tuning curve of the neuron shown in (A). For each orientation, the trial average (circles) ± one standard deviation (shading) of the firing rate (during 1.5 s of stimulation) are plotted. The tuning width, TW, is extracted from a fit to the tuning curve (solid line; see Materials and Methods). The orientation selectivity index [38], OSI = 1−Circular Variance, is computed from the actual data points. (**C**) Same as in (B), for a sample inhibitory neuron. (**D**) Data from 800 excitatory (red) and 200 inhibitory (blue) sample tuning curves, each aligned to the preferred orientation (PO) of its input. The respective tuning curve of the input (same for all neurons) is plotted in green. It is normalized such that it has the same mean value as the output tuning curve. (**E**) Mean (black line) ± standard deviation (gray shading) of output tuning curves, computed from the entire neuron population. The green curve is again the normalized input tuning curve, and the dashed lines indicate the average firing rate for excitatory (red) and inhibitory (blue) populations. OSI and TW are computed from the fit (dotted black line). (**F**) Distribution of the zeroth (F0, baseline) and the second (F2, modulation) Fourier components of output tuning curves in the network. (**G, H**) Distribution of OSI and TW for the population of excitatory (red) and inhibitory (blue) neurons, respectively. The TW distribution is only plotted for neurons with less than 10% error of fit (see Materials and Methods). The distribution of this error is shown in the inset in (H). (**I**) Distribution of the difference between input and output preferred orientations, Δ*PO* = Output PO−Input PO, for excitatory (red) and inhibitory (blue) populations. (**J**) Excitatory (red) and inhibitory (blue) recurrent input to a sample neuron with input preferred orientation, θ* = 0° in response to a stimulus of orientation *θ* = 0°. A sample trace is shown for 200 ms of stimulation, with the distribution for the whole recording time (5 trials of 1.5 s duration) indicated on the right. (**K**) Temporal mean (circles) and standard deviation (bars) of the input for sample excitatory (red) and inhibitory (blue) neurons with different POs (indicated on the x-axis). (**L**) Input tuning curves for the sample neuron shown in (A). Mean and standard deviation (over time) of excitatory (red) and inhibitory (blue) recurrent inputs to the neuron are shown for different stimulus orientations. The feedforward input is shown for comparison (green). (**M**) Input tuning curves for 50 excitatory (red) and 50 inhibitory (blue) sample neurons with different input POs. Feedforward input is again shown in green.
(TIFF)

S2 Fig Orientation selectivity in networks with specific connectivity. **(A–H)** Same as (B–I) in S1 Fig, respectively, for a network with 50% FS connectivity (*μ*_FS_ = 0.5, see Materials and Methods for details) within the excitatory population. In (A) and (B), tuning curves of the same excitatory and inhibitory neurons in the random network (Fig 1B and 1C) are superimposed for comparison (dashed lines). In (D), average tuning curves are plotted separately for excitatory (red) and inhibitory (blue) neurons. (**I–L**) Same as (J–M) in S1 Fig, respectively, for a network with FS connectivity within the excitatory population. (**M**) Mean (solid line) ± one standard deviation (shading) of recurrent input in the network with random connectivity, obtained from 50 excitatory (red) and 50 inhibitory (blue) neurons. (**N**) Same as (M) for the network with FS EE connectivity (*μ*_FS_ = 0.5). (**O**) Tuning of feedforward input compared to the tuning of recurrent excitatory input to excitatory neurons.
(TIFF)

S3 Fig Amplification of input modulation in networks with specific connectivity. (**A**) Eigenvalue spectrum of the normalized weight matrix for a network with random connectivity. The weight matrix is obtained by linearizing the network dynamics and computing neuronal gains (see Materials and Methods for further explanation). The large negative eigenvalue, corresponding to the non-selective mode, is not shown for graphical reasons. It is a result of the inhibition dominance in our networks, and it is responsible for selective attenuation of the common mode. The eigenvalues with second and the third largest magnitude, *λ*_1_ and *λ*_2_, are marked by circles in (B) and (C). The black cross denotes the prediction based on our theory (see Materials and Methods for details). Lower panel: Eigenvectors corresponding to the three largest eigenvalues of the network are plotted versus the input preferred orientation of the corresponding neurons, separately for excitatory and inhibitory neurons (x-axis). The first eigenvector has uniform components and corresponds to the large negative eigenvalue *λ*_0_ (not shown in the spectrum). Only the real part of all components of the eigenvectors are plotted. (**B**) Same as (A), for a network with FS connectivity of E to E synapses. The best fitting cosine function to the second and the third eigenvectors (versus input PO) are shown by solid lines. The difference in the phase of the two cosines (Δ*ϕ*) is indicated in each case.
(TIFF)

S4 Fig Pairwise correlations in networks with different degrees of specific connectivity. (**A**) Distribution of pairwise correlations in the network with random connectivity. Pearson correlation coefficient, CC, of spike counts for different bin sizes (5, 10, 20 and 50 ms, respectively) are computed for randomly sampled neurons (100 excitatory and 100 inhibitory). CC is plotted separately for excitatory-excitatory (EE, red), inhibitory-inhibitory (II, blue), and excitatory-inhibitory (EI, purple) correlations. The mean CC is given for each distribution. (**B**) Same as (A) for networks with an intermediate degree of specific connectivity (50%; *μ*_FS_ = 0.5). At this degree of specific connectivity, the modulation eigenmodes are still stable. (**C**) Same as (A) for networks with a very high degree of FS connectivity (100%; *μ*_FS_ = 1). The modulation eigenmodes are unstable for this network. Note that the distributions show qualitatively the same behavior for different bin sizes. (**D–F**) Distribution of pairwise correlations for all three networks for a very large bin size. It is computed from 200 trials of 500 ms spiking activity, and all neurons with an average firing rate more than 1 spikes/s are included in this analysis.
(TIFF)
