# Correction: Processing of Feature Selectivity in Cortical Networks with Specific Connectivity

**DOI:** 10.1371/journal.pone.0134775

**Published:** 2015-07-31

**Authors:** Sadra Sadeh, Claudia Clopath, Stefan Rotter

The following information is missing from the Funding section: The article processing charge was covered by the open access publication fund of the University of Freiburg.
