# Supplementary figures and images for: Processing of Feature Selectivity in Cortical Networks with Specific Connectivity
Source: PLoS One. 2015 Jun 17;10(6):e0127547. doi: 10.1371/journal.pone.0127547 (PMC4471232; doi:10.1371/journal.pone.0127547)

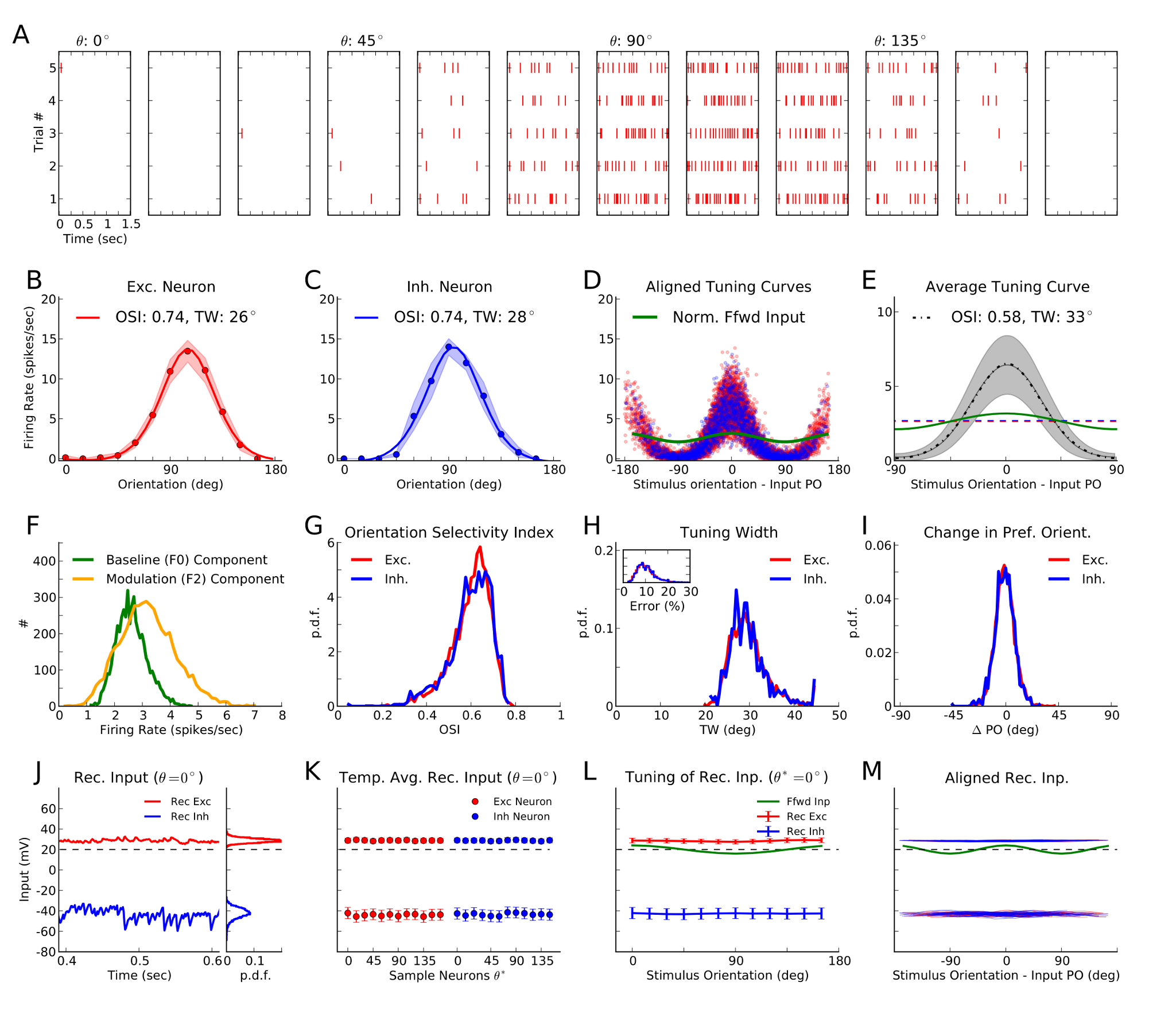

Supplement: S1 Fig — (A) Spikes elicited by a sample excitatory neuron in response to stimuli of different orientations, offered to the network in 5 independent trials. (B) Tuning curve of the neuron shown in (A). For each orientation, the trial average (circles) ± one standard deviation (shading) of the firing rate (during 1.5 s of stimulation) are plotted. The tuning width, TW, is extracted from a fit to the tuning curve (solid line; see Materials and Methods). The orientation selectivity index [38], OSI = 1−Circular Variance, is computed from the actual data points. (C) Same as in (B), for a sample inhibitory neuron. (D) Data from 800 excitatory (red) and 200 inhibitory (blue) sample tuning curves, each aligned to the preferred orientation (PO) of its input. The respective tuning curve of the input (same for all neurons) is plotted in green. It is normalized such that it has the same mean value as the output tuning curve. (E) Mean (black line) ± standard deviation (gray shading) of output tuning curves, computed from the entire neuron population. The green curve is again the normalized input tuning curve, and the dashed lines indicate the average firing rate for excitatory (red) and inhibitory (blue) populations. OSI and TW are computed from the fit (dotted black line). (F) Distribution of the zeroth (F0, baseline) and the second (F2, modulation) Fourier components of output tuning curves in the network. (G, H) Distribution of OSI and TW for the population of excitatory (red) and inhibitory (blue) neurons, respectively. The TW distribution is only plotted for neurons with less than 10% error of fit (see Materials and Methods). The distribution of this error is shown in the inset in (H). (I) Distribution of the difference between input and output preferred orientations, ΔPO = Output PO−Input PO, for excitatory (red) and inhibitory (blue) populations. (J) Excitatory (red) and inhibitory (blue) recurrent input to a sample neuron with input preferred orientation, θ* = 0° in response to a [file pone.0127547.s001.tiff]

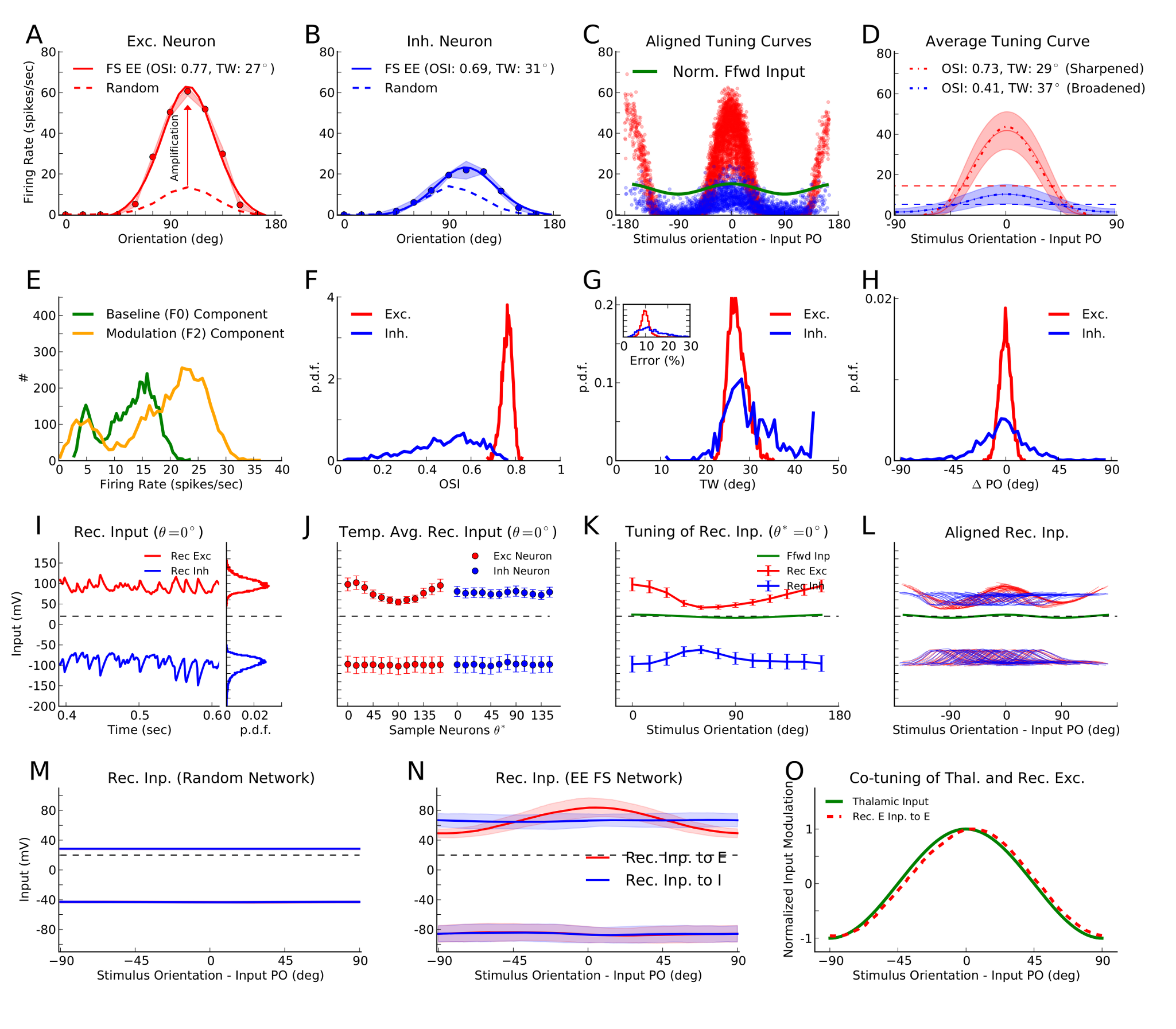

Supplement: S2 Fig — (A–H) Same as (B–I) in S1 Fig, respectively, for a network with 50% FS connectivity (μ FS = 0.5, see Materials and Methods for details) within the excitatory population. In (A) and (B), tuning curves of the same excitatory and inhibitory neurons in the random network (Fig 1B and 1C) are superimposed for comparison (dashed lines). In (D), average tuning curves are plotted separately for excitatory (red) and inhibitory (blue) neurons. (I–L) Same as (J–M) in S1 Fig, respectively, for a network with FS connectivity within the excitatory population. (M) Mean (solid line) ± one standard deviation (shading) of recurrent input in the network with random connectivity, obtained from 50 excitatory (red) and 50 inhibitory (blue) neurons. (N) Same as (M) for the network with FS EE connectivity (μ FS = 0.5). (O) Tuning of feedforward input compared to the tuning of recurrent excitatory input to excitatory neurons. (TIFF) [file pone.0127547.s002.tiff]

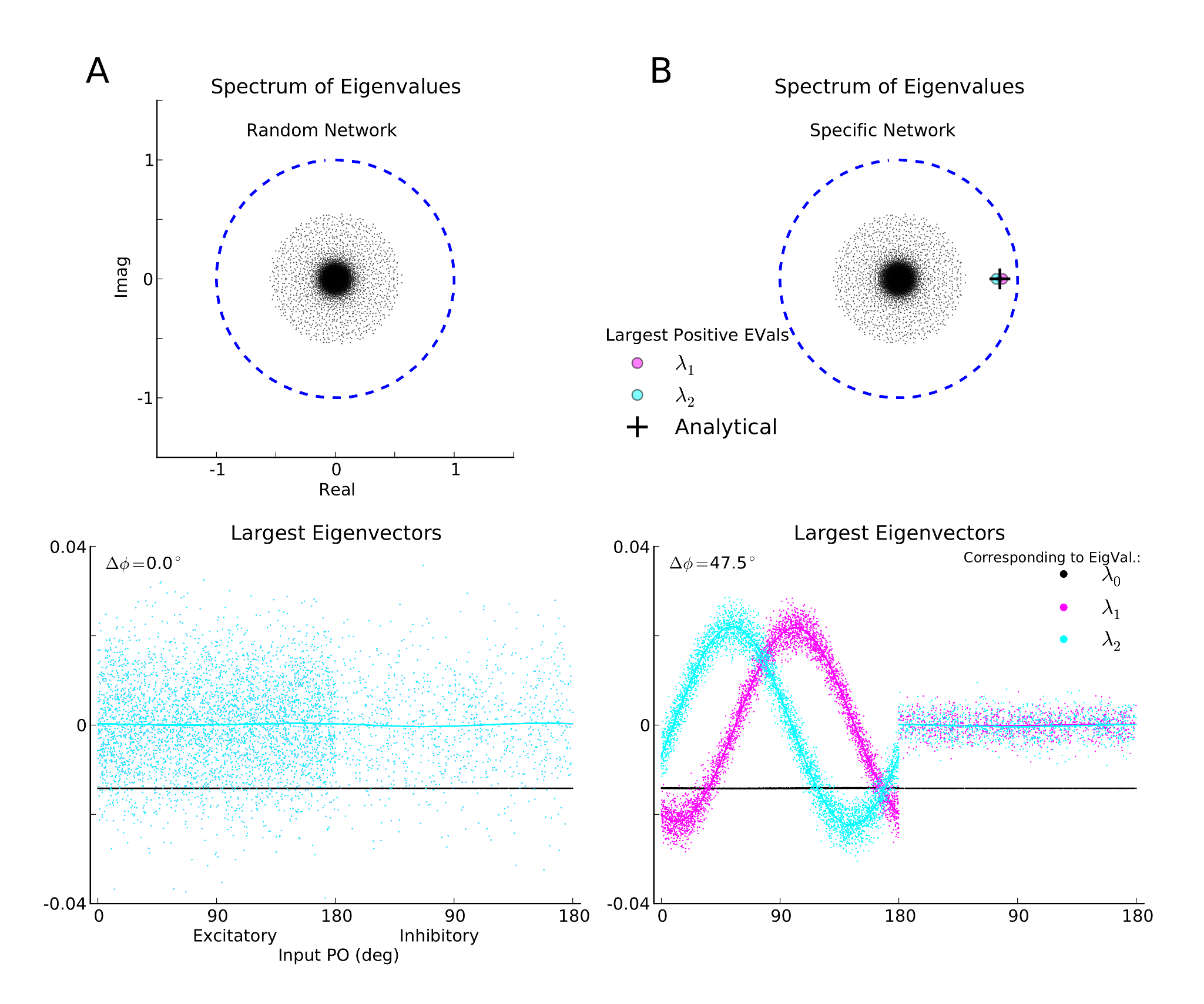

Supplement: S3 Fig — (A) Eigenvalue spectrum of the normalized weight matrix for a network with random connectivity. The weight matrix is obtained by linearizing the network dynamics and computing neuronal gains (see Materials and Methods for further explanation). The large negative eigenvalue, corresponding to the non-selective mode, is not shown for graphical reasons. It is a result of the inhibition dominance in our networks, and it is responsible for selective attenuation of the common mode. The eigenvalues with second and the third largest magnitude, λ 1 and λ 2, are marked by circles in (B) and (C). The black cross denotes the prediction based on our theory (see Materials and Methods for details). Lower panel: Eigenvectors corresponding to the three largest eigenvalues of the network are plotted versus the input preferred orientation of the corresponding neurons, separately for excitatory and inhibitory neurons (x-axis). The first eigenvector has uniform components and corresponds to the large negative eigenvalue λ 0 (not shown in the spectrum). Only the real part of all components of the eigenvectors are plotted. (B) Same as (A), for a network with FS connectivity of E to E synapses. The best fitting cosine function to the second and the third eigenvectors (versus input PO) are shown by solid lines. The difference in the phase of the two cosines (Δϕ) is indicated in each case. (TIFF) [file pone.0127547.s003.tiff]

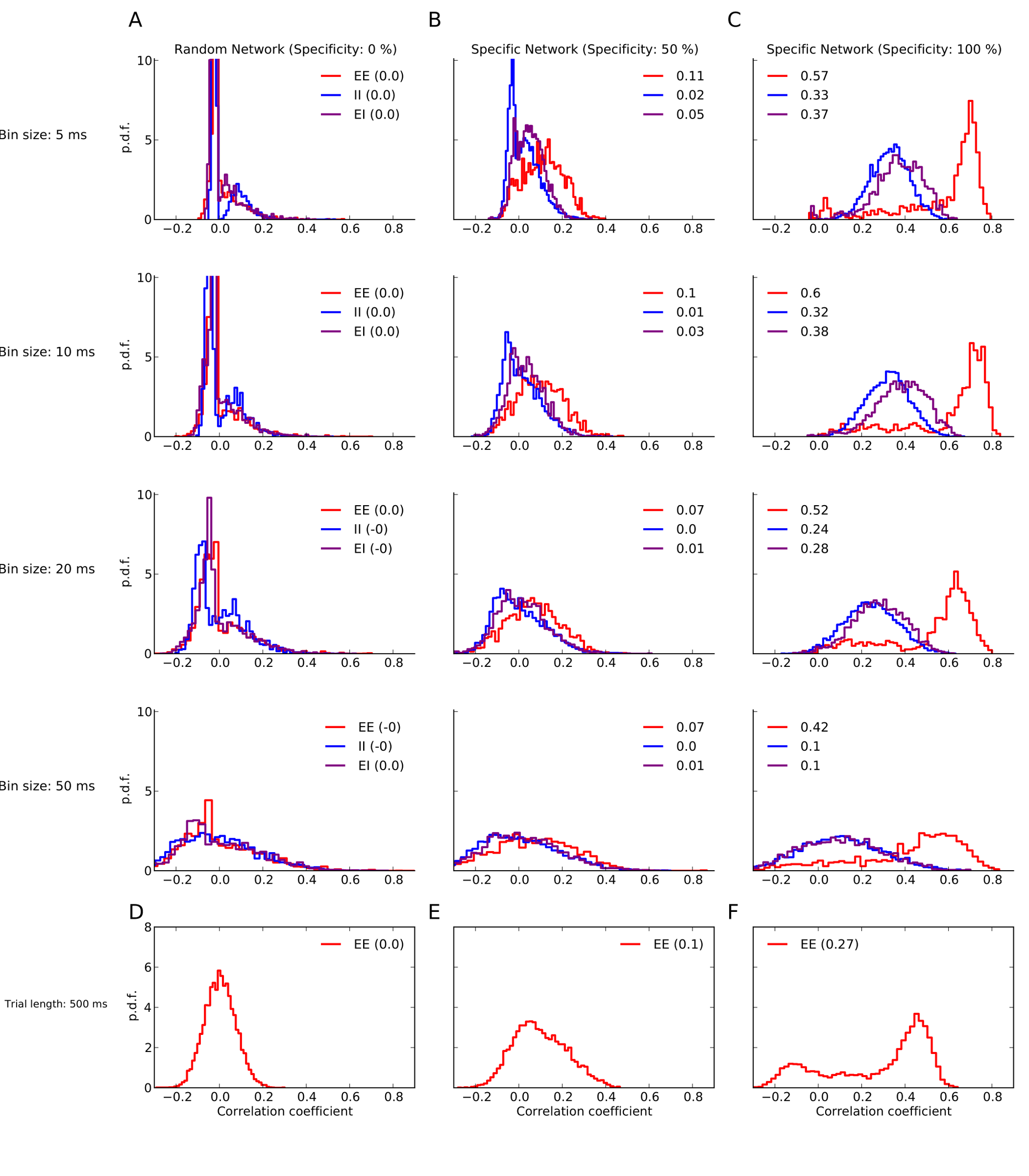

Supplement: S4 Fig — (A) Distribution of pairwise correlations in the network with random connectivity. Pearson correlation coefficient, CC, of spike counts for different bin sizes (5, 10, 20 and 50 ms, respectively) are computed for randomly sampled neurons (100 excitatory and 100 inhibitory). CC is plotted separately for excitatory-excitatory (EE, red), inhibitory-inhibitory (II, blue), and excitatory-inhibitory (EI, purple) correlations. The mean CC is given for each distribution. (B) Same as (A) for networks with an intermediate degree of specific connectivity (50%; μ FS = 0.5). At this degree of specific connectivity, the modulation eigenmodes are still stable. (C) Same as (A) for networks with a very high degree of FS connectivity (100%; μ FS = 1). The modulation eigenmodes are unstable for this network. Note that the distributions show qualitatively the same behavior for different bin sizes. (D–F) Distribution of pairwise correlations for all three networks for a very large bin size. It is computed from 200 trials of 500 ms spiking activity, and all neurons with an average firing rate more than 1 spikes/s are included in this analysis. (TIFF) [file pone.0127547.s004.tiff]
